# OP48 A Comprehensive Framework For Implementing Decentralized Health Care, Informed By The Experiences Of Four Benchmark Countries

**DOI:** 10.1017/S0266462325101062

**Published:** 2025-12-29

**Authors:** Priscilla Loze, Natalia Ramos, Juliana Simoes, Tamie Martins, Manuel Andres Schepp

## Abstract

**Introduction:**

National healthcare systems face challenges, including rising costs, aging populations, and increasing rates of chronic diseases, especially in rural and underserved areas. Decentralized care (DC) offers healthcare services in less complex settings, reducing travel, optimizing resource use, and enhancing patient centricity. This study explored DC initiatives, implementation strategies, challenges, and outcomes in four countries to establish a scalable framework.

**Methods:**

Four countries with strong policies and initiatives supporting decentralized care were selected (Belgium, the Netherlands, Singapore, the UK) to understand how governments can enhance clinical outcomes, optimize costs, and improve patient satisfaction through DC models. Data collection involved a review of government documents and reports, complemented by interviews with senior stakeholders from hospital systems and government bodies. The analysis focused on understanding the principles and practice of DC in these countries.

**Results:**

Significant progress was observed in these countries. The UK’s integrated care providers achieved a 12 percent reduction in hospital admissions, while Singapore’s MIC@Home saved 7,000 bed days and increased telemedicine usage by 40 percent. The Netherlands’ Better@Home saved EUR2 million annually and increased remote care by 20 percent. Belgium’s model reduced heart failure readmissions by 15 percent and improved rural access and appointment satisfaction by seven percent. A common framework emerged, structured around four central pillars: robust policies and regulations, advanced technology and data integration, development of infrastructure and community care, and comprehensive training for healthcare professionals.

**Conclusions:**

DC is a pivotal and transformative approach that can enhance patient care and ensure sustainability of health systems, despite implementation complexities. Success hinges on robust policies, technological infrastructure, data integration, and adaptive training mechanisms. These benchmarks underscore the importance of collaboration among multiple stakeholders to implement DC, in order to sustainably meet the evolving demands and provide patient-centered care.

